# Non-contact REM/NREM sleep staging from piezoelectric signals using respiratory and body-movement features with auxiliary TWED-based respiratory stability measures

**DOI:** 10.3389/fdgth.2026.1780166

**Published:** 2026-06-15

**Authors:** Shaonan Wang, Jia Yu, Xianjun Yang, Diming Liu, Qingyuan Bai, Jiakuai Yu, Shuai Ding, Yang Xu, Daomin Zhu

**Affiliations:** 1 Hefei Institutes of Physical Science, Chinese Academy of Sciences, Hefei, China; 2 University of Science and Technology of China, Hefei, China; 3 School of Nursing, Bengbu Medical University, Bengbu, China; 4 Hefei Fourth People's Hospital, Hefei, China

**Keywords:** body-movement, piezoelectric signals, respiratory pattern stability, respiratory variability, sleep stage classification, time warp edit distance, XGBoost

## Abstract

**Introduction:**

Non-contact sleep monitoring based on under-mattress piezoelectric sensing is attractive for low-burden home use, but REM/NREM discrimination remains challenging. This study aimed to investigate whether respiratory pattern stability, quantified by Time Warp Edit Distance (TWED)-based respiratory interval sequence (RIS) similarity features, could improve discrimination between the two major sleep states within sleep itself.

**Methods:**

Overnight piezoelectric and polysomnography (PSG) data were collected simultaneously from 85 clinical subjects. PSG-labeled wake epochs were excluded, and the task was formulated as binary REM/NREM classification. From piezoelectric signals, we extracted conventional body-movement features, respiratory variability features, and multi-scale TWED-based RIS similarity features. Feature normalization was performed within each subject using the full-night unlabeled feature distribution, consistent with the intended *post-hoc* whole-night analysis scenario. An XGBoost classifier was evaluated using subject-wise nested leave-one-out cross-validation (LOOCV), with the REM-continuity fusion threshold selected within each training fold by inner subject-grouped 5-fold cross-validation. Respiratory signal extraction was additionally validated against PSG airflow in resting and full-night tests.

**Results:**

Feature normalization improved performance across all feature sets. The best result was achieved by combining conventional body-movement and respiratory variability features with TWED-based RIS similarity features, yielding an accuracy of 84.39 ± 12.76%, Cohen's Kappa of 0.524 ± 0.241, REM precision of 0.600 ± 0.210, REM recall of 0.735 ± 0.226, and REM F1-score of 0.603 ± 0.211 under nested LOOCV. Compared with the normalized conventional feature set without RIS similarity, adding the TWED-based features improved both Kappa and REM F1-score. In respiratory tests, PVDF-derived respiration showed low detection error and good agreement with the airflow reference under both posture-change and overnight conditions.

**Discussion:**

These findings indicate that TWED-based RIS similarity features provide useful complementary information beyond conventional descriptors and support the feasibility of using respiratory pattern stability derived from non-contact piezoelectric signals for within-sleep REM/NREM classification. At the current level of performance, the proposed method is better viewed as a low-burden adjunctive tool for offline whole-night longitudinal monitoring and trend assessment in home-like settings rather than as a replacement for PSG-based clinical diagnosis, real-time sleep staging, or full sleep-stage scoring.

## 1 Introduction

Sleep staging is essential for assessing sleep quality, characterizing sleep architecture, and supporting the diagnosis and management of sleep disorders. In standard polysomnography (PSG)-based scoring, a full night of recording is conventionally annotated as wakefulness (WAKE), rapid eye movement sleep (REM), and non-rapid eye movement sleep (NREM), with NREM further divided into N1, N2, and N3. Manual sleep staging based on electroencephalography (EEG), electrooculography (EOG), and electromyography (EMG) remains the clinical gold standard. However, PSG is expensive, labor-intensive, and dependent on specialized equipment and trained technicians in clinical environments (1). In addition, the burden of electrode attachment and overnight instrumentation may disturb natural sleep, limiting the practicality of PSG for repeated or long-term home monitoring (2).

To address these limitations, unobtrusive sleep monitoring technologies have been increasingly explored for home use (3). Among them, under-mattress piezoelectric sensing is particularly attractive (4) because it is contactless, low-cost, easy to install, and capable of continuously capturing minute mechanical vibrations caused by respiration, body movement, and, to a lesser extent, cardiac activity. In practice, two classes of information are most reliably derived from piezoelectric signals for sleep analysis: body-movement activity and respiratory dynamics. This emphasis is partly methodological—piezoelectric signals are often noise-prone and heartbeat-related features are difficult to capture robustly across subjects—but also physiological. Body movement reflects postural shifts, micro-arousals, and changes in muscle tone, whereas respiratory dynamics reflect autonomic regulation across sleep (5–8). These signals therefore provide a principled basis for sleep-stage discrimination in non-contact settings.

From a physiological perspective, REM and NREM sleep differ in ways that are particularly relevant to piezoelectric sensing. NREM sleep is generally associated with more stable autonomic regulation and more regular breathing, especially as sleep deepens, whereas REM sleep is characterized by greater respiratory irregularity, fluctuating autonomic activity, and markedly reduced skeletal muscle tone (9, 10). Thus, the distinction between REM and NREM is not only reflected in whether breathing is fast or slow, but also in how stable and repeatable the respiratory pattern remains over time. In the present study, PSG recordings were originally scored into WAKE, REM, N1, N2, and N3 according to the AASM (American Academy of Sleep Medicine) scoring criteria. For the purpose of this work, N1, N2, and N3 were merged into a single NREM category, and WAKE epochs were excluded so that the task specifically focused on REM-versus-NREM discrimination within sleep. This binary formulation was chosen because the primary objective of this study was to investigate whether respiratory pattern stability can improve discrimination between the two major sleep states within sleep itself, rather than to perform full-stage sleep scoring. The present study focuses on within-sleep discrimination between PSG-labeled REM and NREM epochs. Our working hypothesis is that respiratory pattern stability, as characterized by respiratory interval sequence (RIS)-based features, differs between REM and NREM sleep epochs, rather than across the full set of nocturnal states.

A variety of non-contact and low-burden approaches to sleep analysis have been reported, differing in sensing modality, feature design, and classification strategy (11). Kambayashi et al. used an infrared motion sensor to estimate body-movement density and showed that it was related to sleep-stage transitions (12). Long et al. extracted amplitude-based respiratory features from PSG and, after subject-specific normalization, reported 76.2% accuracy and a Cohen's Kappa of 0.45 for WAKE/REM/NREM classification (13). Samy et al. used pressure-sensitive textile sheets to capture respiration and body movement and achieved 72.2% accuracy on WAKE/REM/NREM classification with a naive Bayes classifier (14). Studies focusing specifically on binary REM/NREM discrimination are fewer. Walch et al. used Apple Watch accelerometry and heart-rate–related features with a multilayer perceptron (MLP), obtaining 72% accuracy (15). Jaworski et al. combined ballistocardiography (BCG) with Apple Watch signals and used nonlinear heartbeat features with a long short-term memory (LSTM) model, achieving 72.7% accuracy against PSG (16). Despite these advances, non-EEG approaches still generally underperform PSG, and multi-stage classification frameworks may dilute features that are especially informative for distinguishing REM from NREM.

Many existing non-contact approaches rely on body-movement intensity, respiratory rate, or conventional respiratory variability descriptors. Although these features are useful, they may not fully capture the short-term temporal stability of respiratory control across neighboring epochs. We therefore hypothesize that a more fundamental distinction between REM and NREM lies in respiratory pattern stability: under relatively stable autonomic control, NREM breathing tends to be more rhythmic and repeatable, whereas REM breathing is more irregular and less predictable due to phasic neural activity and autonomic fluctuation. To quantify this property, we introduce a similarity-based representation of respiratory interval sequences (RIS) using Time Warp Edit Distance (TWED) (17), a time-series metric that explicitly accounts for both value differences and temporal misalignment. By measuring the similarity between RIS in adjacent windows, TWED provides a way to characterize short-term respiratory pattern stability rather than only within-window variability.

**Figure 1:**
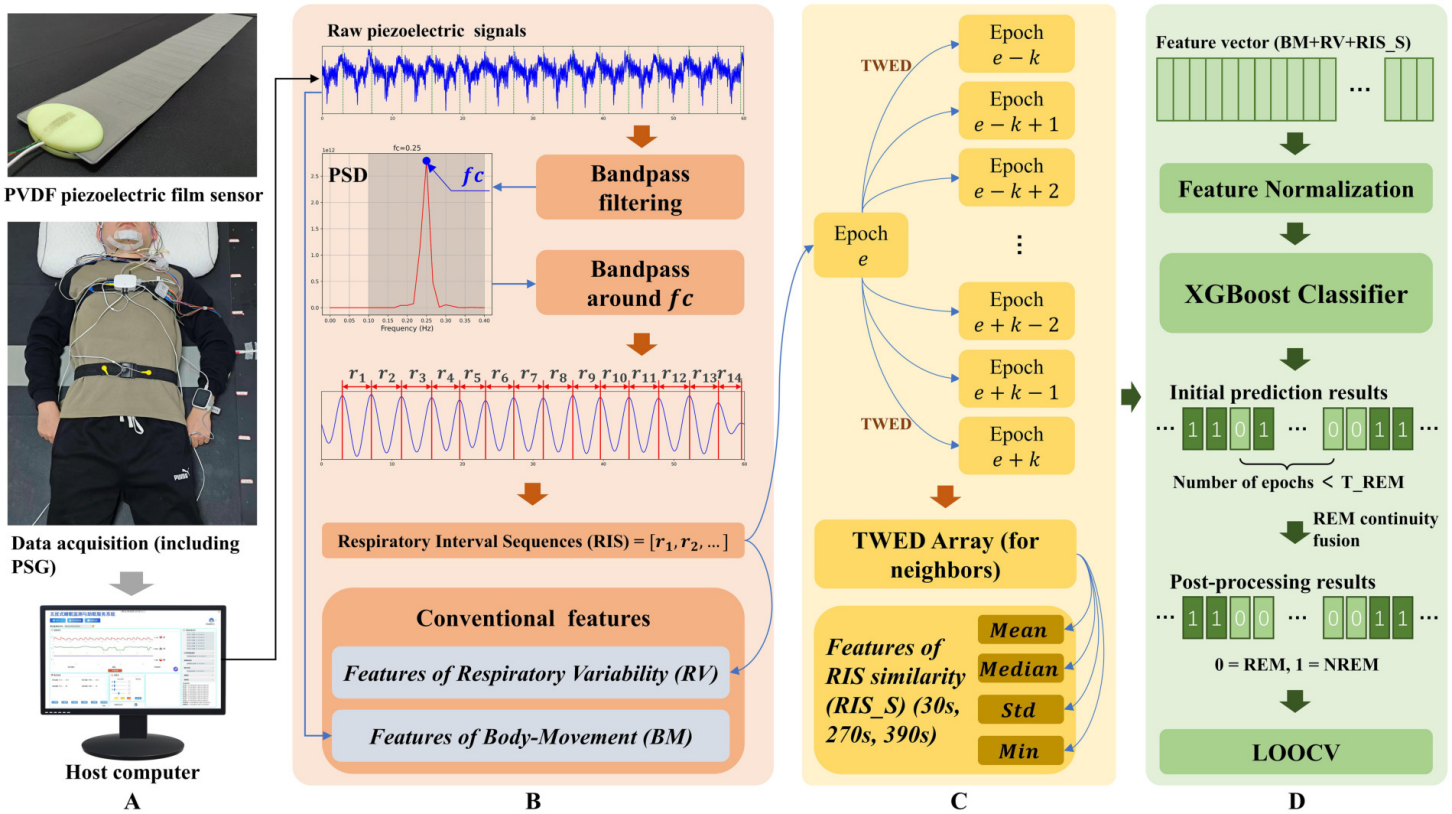
Overview of the proposed framework for REM/NREM sleep staging from non-contact piezoelectric sensing. **(A)** Non-contact piezoelectric sensing and PSG reference; **(B)** Calculation of RIS and extraction of conventional body-movement and respiratory variability features (for brevity, time variables are ignored in RIS); **C.** Extraction of RIS similarity features; **D.** Model training and validation (including post-processing rules).

Accordingly, this study proposes a non-contact REM/NREM sleep staging method using body-movement and respiratory features extracted from piezoelectric signals. The overall framework is shown in Figure 1 and includes data acquisition, RIS calculation, extraction of conventional body-movement and respiratory variability features, extraction of TWED-based RIS similarity features, and model training and validation. The main contribution of this work is the design and implementation of a set of multi-scale TWED-based RIS similarity features as auxiliary biomarkers of respiratory pattern stability. Using a dataset of 85 clinical subjects, we systematically compare model performance with and without these features. The proposed approach is intended as a low-burden method for *post-hoc* whole-night REM/NREM discrimination from completed overnight recordings, which may support unobtrusive longitudinal sleep monitoring and night-to-night trend assessment while not replacing PSG-based clinical diagnosis.

The remainder of this paper is organized as follows. Section 2 describes the data acquisition system and the proposed method. Section 3 presents the experimental results. Section 4 discusses the findings, limitations, and potential use-cases. Section 5 concludes the study.

## 2 Materials and methods

### 2.1 Data acquisition

We deployed a custom piezoelectric sensing system (Figure 1A) for non-invasive signal acquisition, comprising a 100 cm × 15 cm × 2 mm PVDF (polyvinylidene fluoride) piezoelectric film sensor placed 40 cm from the head under the mattress, a master control unit, and a custom-built analog front-end. Because the PVDF sensor produces high-impedance charge signals, the front-end was implemented as a discrete signal-conditioning circuit rather than using a dedicated commercial AFE chip. Specifically, the circuit consisted of three stages: a charge amplification stage, an inverting amplification stage, and an analog-to-digital conversion stage. After amplification, the piezoelectric signal was digitized at a sampling rate of 300 Hz. Continuous signal data were then transmitted via TCP/IP to a WPF (Windows Presentation Foundation)-based host computer application for packet parsing, local storage, and command communication. This design was intended to preserve weak respiration-related vibrations while providing sufficient resolution and dynamic range for overnight monitoring.

The dataset used in this study was collected from 85 subjects recruited from the Sleep Department of Hefei Fourth People's Hospital. Piezoelectric signals were recorded continuously overnight, while multi-channel PSG signals were acquired simultaneously. The PSG setup included standard EEG, EOG, chin EMG, ECG, and a respiratory airflow channel, which was used as the reference for respiratory-rate comparison. PSG recordings were manually scored into WAKE, REM, N1, N2, and N3 according to the AASM criteria by three clinicians, and discrepancies were resolved by consensus. For the present binary classification task, only PSG-labeled sleep epochs were retained. Specifically, 30-s epochs scored as REM or NREM were included in the analysis, whereas wake epochs, including wake after sleep onset (WASO), were excluded before model training and performance evaluation. NREM stages were merged into a single class for the binary REM/NREM task. Piezoelectric data were aligned with polysomnographic data by timestamp and segmented into 30-second windows. All subjects voluntarily participated in the data collection, and the data collection adhered to ethical standards. Table 1 summarizes the demographic and REM/NREM sleep stage statistics of the participants [mean ± standard deviation (SD) and range].

**Table 1:** Subjects' demographics and REM/NREM sleep stage statistics.

Variable	Mean ± SD	Range
Sex	38 males and 47 females	
Age (Years)	34.7 ± 16.8	10–70
BMI^a^ (kg/m2)	22.9 ± 4.4	14.7–42.2
TRT^b^ (minutes)	461.5 ± 70.0	155.0–574.0
REM (%)	14.4 ± 7.2	0.4–36.1
NREM (%)	72.1 ± 9.6	46.5–94.2

The percentage of REM and NREM refers to the proportion of each stage in a full night's sleep.

a Body mass index.

b Total recording time.

To further characterize the clinical composition of the cohort, we additionally grouped subjects according to pre-monitoring diagnosis and respiratory event burden. Based on the pre-monitoring diagnosis, subjects were consolidated into three broad categories: sleep-disorder-related, psychiatric/psychological-disorder-related, and other/unspecified. The sleep-disorder-related category included narcolepsy, hypersomnia, hypersomnia under evaluation, rapid eye movement sleep behavior disorder (RBD), sleepwalking, nightmare disorder, insomnia, and compound diagnoses containing any of these conditions. The psychiatric/psychological-disorder-related category included anxiety disorders, depressive disorders, bipolar/affective disorders, and schizophrenia-spectrum disorders. Subjects with unspecified diagnoses or diagnoses that could not be reliably classified were assigned to the other/unspecified category. In addition, subjects were stratified according to apnea–hypopnea index (AHI) as AHI < 5 and AHI ≥ 5 events/h. The resulting distribution of the cohort is summarized in Table 2.

**Table 2:** Distribution of diagnosis-defined categories across AHI strata in the study cohort.

AHI group	Sleep-disorder-related	Psychiatric/psychological-disorder-related	Other/unspecified	Total
AHI < 5	24	35	5	64
AHI ≥ 5	3	12	6	21

### 2.2 Respiratory signal extraction and assessment

#### 2.2.1 Signal preprocessing and segmentation

**Figure 2:**
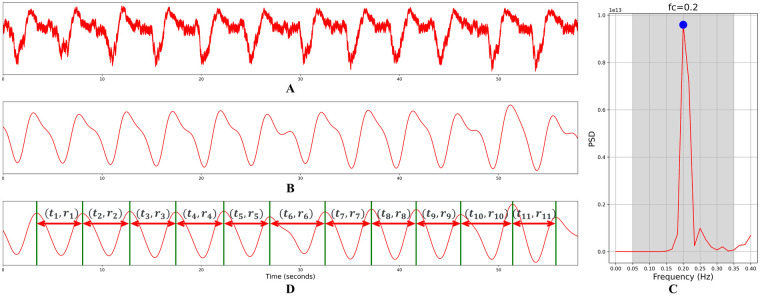
Taking 58s signals as an example to illustrate the calculation process of RIS. **(A)** Raw piezoelectric signals; **(B)** The signal obtained after filtering the raw piezoelectric signals using a 3rd-order Butterworth bandpass filter (0.15–0.4 Hz); **(C)** The power spectrum density (PSD) of **(B)**, where the frequency corresponding to the blue point is the fc, and the gray shadow represents the frequency band of 0.15 Hz before and after the fc; **(D)** The signal obtained after filtering the raw piezoelectric signals using a bandpass filter with a range of [fc−0.15,fc+0.15], where the red vertical line represents the detected respiratory peak position, $r_{i}$ represents the time difference between two respiratory peaks, and $t_{i}$ is a time variable representing the time elapsed from the start of the first respiratory interval of this epoch to the end of the i-th respiratory interval (i=1,2,3,…).

To identify the RIS and extract respiratory-related features, the raw piezoelectric signals is first filtered using a 3rd-order Butterworth bandpass filter (0.15–0.4 Hz). The Welch method is then applied to calculate the power spectral density, and the frequency with maximum power in this range is identified as the respiratory dominant frequency (fc). A second filtering is performed to raw piezoelectric signals using a bandpass filter with a range of [fc−0.15,fc+0.15], resulting in a smooth signal with a clear respiratory waveform. RIS are obtained by detecting peaks and calculating time differences between them. Figure 2 illustrates the calculation process of RIS using 58s signals as an example.

A low-pass filter with a 0.5 Hz cutoff was applied to the Airflow channel from PSG to obtain a smooth curve, with peak detection used to calculate the respiratory interval as the gold standard. The average respiratory rate was calculated every 30 s for both piezoelectric and Airflow signals. Two sliding windows of 9 and 13 epochs were applied to extract features for each sleep stage label, as research (18) suggests that longer sequences improve sleep stage classification.

#### 2.2.2 Respiratory tests

To assess the robustness of respiratory signal extraction from a single PVDF film sensor under posture changes and overnight sleep conditions, we conducted two validation tests, including a resting test and a full-night sleep test. 10 subjects were randomly selected from the 85-subject cohort, including 5 subjects with Sleep-Disordered Breathing (SDB) and 5 subjects without SDB.

In the resting test, each subject underwent 30 min of simultaneous PVDF and PSG recording, including 10 min in the supine, 10 min in the left lateral, and 10 min in the right lateral. Except for the required posture changes, subjects were instructed to remain as still as possible. In the full-night sleep test, PVDF-derived respiratory signals were compared with the Airflow from PSG over the entire overnight recording.

Based on the method described in Section 2.2.1, we located respiratory peaks from the synchronously acquired PVDF and Airflow signals, respectively. Due to the phase difference between respiratory airflow and chest respiratory motion, the PVDF respiratory peak closest to each Airflow respiratory peak within a ± 0.5 s window was assigned to that Airflow respiratory peak. Unassigned PVDF respiratory peaks were considered false positives, and Airflow respiratory peaks not assigned to any PVDF respiratory peak were considered false negatives. For each subject in the respiratory tests, we calculated the false-positive rate (FPR), false-negative rate (FNR), and the mean error of respiratory rate computed over 30-s windows (BR30). Agreement between PVDF-derived respiratory rate and the reference respiratory rate was further evaluated using correlation analysis and Bland–Altman analysis.

### 2.3 Feature extraction

#### 2.3.1 Conventional body-movement and respiratory variability features

(1) Features of Body-Movement
  For raw piezoelectric signals of 30s (9,000 sampling points), it is first segmented into 5s (1,500 sampling points) windows, and a total of 6 non-overlapping sub-segments are obtained. The absolute value of the signal amplitude of the i-th sub-segment (denoted as $x_{i}$) is taken and summed, and multiplied by the time step of 0.5s (custom value) to obtain the activity volume of the 5s window, that is,
  $$M_{i}=0.5\overset{1500}{\underset{\;j=1}{\sum }}|x_{i}[j]|,\;i=1,2,\ldots ,6$$

The definition of activity volume here refers to the calculation process of Riemann integration (19). Let $\&\mathrm{lcub};M_{1},\;M_{2},\ldots ,M_{6}\&\mathrm{rcub};$ be the activity volume list of all sub-segments, let $M_{min}=\min\&\mathrm{lcub};M_{1},M_{2},\ldots ,M_{6}\&\mathrm{rcub};$, then the Body-Movement Intensity Index (*BII*) is defined as:

$$BII=\frac{1}{6}\overset{6}{\underset{i=1}{\sum }}(M_{i}-M_{min})$$

By subtracting the minimum activity volume from the activity volume in each 5-s subsegment and taking the average, BII can reflect the level of change in activity volume relative to the lowest activity intensity subsegment within a 30-s window, thereby quantifying the subject's physical activity.

In order to finely detect the subtle limb changes of subjects in adjacent epochs, we calculated two derived features based on BII. Assuming that BII of the current epoch (index i) is *BII_i_*, the two derived features (*BIIDF*) are defined as follows:

$$BIIDF_{1}=\underset{k}{\sum }|BII_{k}-BII_{k-1}|\;k=\{i-1,i,i+1,i+2\}$$

$$BIIDF_{2}=\sqrt{\frac{1}{5}\underset{k}{\sum }{\left(BII_{k}-\bar{BII}\right)}^{2}}\;k=\{i-2,i-1,i,i+1,i+2\}$$

*BIIDF*_1_ represents the cumulative difference of BII of the five adjacent 30s epochs, and $BIIDF_{2}$ represents the standard deviation of BII of the five adjacent 30s epochs.

When the body-movement event occurs, the intuitive change in the piezoelectric signals is a change in amplitude (in fact, BII and its derivative features also originate from this). Here we consider two additional amplitude-based features in order to more accurately reflect the changes in the amplitude of the piezoelectric signals when the body-movement event occurs within a single 30s epoch. Assuming that the piezoelectric signals of a 30s epoch is s(n), where *n* represents the sampling point index, the two amplitude features (AF) are defined as follows:

$$AF_{1}=\frac{1}{N}\overset{N}{\underset{n=1}{\sum }}|s(n)|,\;N=9000$$

$$AF_{2}=\overset{N}{\underset{n=1}{\sum }}s(n{)}^{2},\;N=9000$$

*AF*_1_ represents the mean value of the piezoelectric signal amplitude within the 30s epoch, and $AF_{2}$ represents the sum of the squares of the piezoelectric signal amplitude within the 30s epoch.

(2) Features of Respiratory Variability
  Referring to heart rate variability, we call the indicators describing the fluctuation of respiratory pattern extracted from the changes of RIS in time-domain and frequency-domain as features of respiratory variability. They usually reflect the irregularity, periodicity, etc. of the respiratory process. This study used time-frequency domain analysis methods to extract a series of time-domain features and frequency-domain features from the RIS obtained in Section 2.2. The detailed calculation methods of these features are similar to those of heart rate variability, and are only qualitatively described in this study. The time-domain features include the mean, standard deviation, root mean square difference, median, root mean square, nn50 and pnn50 of the RIS. The fast Fourier transform was performed on the RIS, and the spectrum of the RIS was divided into the following segments: very low frequency (0.01–0.05 Hz, vlf), low frequency (0.05–0.15 Hz, lf) and high frequency (0.15–0.5 Hz, hf). Then the frequency-domain features of the RIS were calculated based on each frequency band, including the total power density spectrum, very low frequency component, low frequency component, high frequency component and lf/hfratio. In addition, we extracted two respiratory features, Cm (20) and ERMRB (21), which have been used for sleep stage classification in previous studies. Further description of these features is shown in Table 3. The 30s sequence is mainly used to extract time-domain features to reflect short-term respiratory variability. The 270s and 390s sequence covers two types of features to provide more accurate context information (given that the small number of respiratory intervals in 30 s sequences makes them unsuitable for frequency-domain analyses).

**Table 3:** Summary of RIS features.

Feature Type	Feature Name	Description	Calculation Object
Time-domain features	mean	Mean of RIS.	30s RIS, 270sRIS and 390s RIS
	std	Standard deviation of RIS.	
	rmssd	The root mean square of the difference between adjacent respiratory intervals.	
	median	Median of RIS.	
	rms	Root mean square of RIS.	
	nn50	The number of times the difference between adjacent respiratory intervals which is greater than 50 milliseconds.	
	pnn50	The proportion of difference between adjacent respiratory intervals which is greater than 50 milliseconds.	
Frequency-domain features	totalpower	Total power of the RIS spectrum.	270s RIS and 390s RIS
	vlf	The power in the frequency range of 0.01–0.05 Hz, which mainly reflects the low-frequency fluctuations of the autonomic nervous system.	
	lf	The power in the frequency range of 0.05–0.15 Hz, which is associated with sympathetic nerve activity.	
	hf	The power in the frequency range of 0.15–0.5 Hz, which is associated with parasympathetic activity.	
	ratiooflfandhf	The ratio of low-frequency power components to high-frequency power components, which is used to measure the balance between sympathetic and parasympathetic nerve activity.	
Features proposed in previous studies	Cm (20)	The mean of respiration-by-respiration correlations.	30s RIS
	ERMRB (21)	The energy ratio of main respiratory band in the frequency-domain.	
Similarity features	$D_{mean}$	The mean of the RIS similarity array between the current epoch and the adjacent epochs.	30s RIS, 270sRIS and 390s RIS
	$D_{median}$	The median of the RIS similarity array between the current epoch and the adjacent epochs.	
	$D_{std}$	The standard deviation of the RIS similarity array between the current epoch and the adjacent epochs.	
	$D_{min}$	The minimum of the RIS similarity array between the current epoch and the adjacent epochs.	

#### 2.3.2 Features based on similarity measurement of RIS

To quantify short-term stability of respiratory control across sleep, we compute the similarity between the current epoch's respiratory interval sequence (RIS) and those of its neighboring epochs using Time Warp Edit Distance (TWED) (17). The resulting statistics form a feature vector that complements conventional respiratory variability and body-movement descriptors for REM/NREM discrimination.

Let the analysis unit be an epoch *e* (duration aligned with the staging window). The RIS of epoch *e* is

$$R^{\left(e\right)}=\{\left(t_{i}^{\left(e\right)},r_{i}^{\left(e\right)}\right){\}}_{i=1}^{n_{e}},$$

where $r_{i}^{\left(e\right)}$denotes the i-th inter-breath interval (in milliseconds) and $t_{i}^{\left(e\right)}$the corresponding timestamp (in milliseconds).

For a set of neighboring epochs $N_{k}=\&\mathrm{lcub};-k,\ldots ,-1,1,\ldots ,k\&\mathrm{rcub};$ around *e* (neighborhood size k), let $R^{\left(e+p\right)}$ denote the RIS of epoch e+p.

TWED measures dissimilarity between two time-stamped sequences by combining value differences and temporal misalignment while permitting insertion/deletion operations. For sequences $R^{\left(a\right)}=\&\mathrm{lcub};(t_{i}^{\left(a\right)},r_{i}^{\left(a\right)})\&\mathrm{rcub}{;}_{i=1}^{n_{a}}$ and $R^{\left(b\right)}=\&\mathrm{lcub};(t_{j}^{\left(b\right)},r_{j}^{\left(b\right)})\&\mathrm{rcub}{;}_{\;j=1}^{n_{b}}$, TWED is computed via a dynamic-programming recursion on D(i,j):

$$D(0,0)=0,\;D(i,0)=\infty ,\;D(0,j)=\infty ,\;r_{0}^{\left(a\right)}=r_{0}^{\left(b\right)}=0,\;t_{0}^{\left(a\right)}=t_{0}^{\left(b\right)}=0$$

$$D(i,j)=\min\{\underset{match}{\underbrace{D(i-1,j-1)+|r_{i}^{\left(a\right)}-r_{j}^{\left(b\right)}|+\upsilon |t_{i}^{\left(a\right)}-t_{j}^{\left(b\right)}|}},$$

$$\;\;\underset{delete\;in\;a}{\underbrace{D(i-1,j)+|r_{i}^{\left(a\right)}-r_{i-1}^{\left(a\right)}|+\upsilon |t_{i}^{\left(a\right)}-t_{i-1}^{\left(a\right)}|+\lambda }},$$

$$\;\;\underset{delete\;in\;b}{\underbrace{D(i,j-1)+|r_{j}^{\left(b\right)}-r_{\;j-1}^{\left(b\right)}|+\upsilon |t_{j}^{\left(b\right)}-t_{\;j-1}^{\left(b\right)}|+\lambda }}\}$$

and $d_{\mathrm{TWED}}(R^{\left(a\right)},R^{\left(b\right)};\lambda ,\nu )=D(n_{a},n_{b})$. Here, λ>0 is the edit (penalty) controlling tolerance to insertions/deletions, and υ≥0 is the time-stiffness weighting temporal misalignment. To avoid additional hyper-parameterization and reduce overfitting risk on the 85-subject cohort, the edit penalty and time-stiffness were fixed *a priori* to λ=1.0 and ν=0.001 across all experiments. A small sensitivity check on the dataset (± 50% perturbation of either parameter) produced no material change in validation accuracy/ Cohen's Kappa; therefore, the fixed values were used throughout.

For each epoch *e* and each time scale *s* (we consider 30 s, 270 s, and 390 s RIS constructed by aggregating breaths within the corresponding windows), we compute TWED to *k* neighboring epochs:

$${\;D}^{\left(e,s\right)}=[d_{TWED}(R^{\left(e,s\right)},R^{\left(e+p,s\right)};\lambda ,\upsilon ){]}_{\;p\in N_{k}},$$

where $t_{i}$ is constructed at each scale using the intrinsic timestamp of the corresponding window, without cross-window splicing. Considering the trade-off between computational efficiency and classification accuracy, we set k to 5, that is, for each epoch, we calculate the similarity features between its RIS and those of the 10 adjacent epochs. To obtain a compact summary of short-term “pattern stability”, we aggregate each ${\;D}^{\left(e,s\right)}$ into four conventional statistics:

$$stats(D^{\left(e,s\right)})=[D_{mean},D_{median},D_{std},D_{min}].$$

Supplementary Appendix Figure S1 illustrates the extraction process of RIS similarity (RIS_S) features using a 30s epoch as an example. The complete RIS_S feature vector is obtained by concatenating the statistics across scales:

$$RIS\_S_{\left(e\right)}\;=[stats(D^{\left(e,30\right)}),\;stats(D^{\left(e,270\right)}),\;stats(D^{\left(e,390\right)}\;)].$$

The computational complexity is $O(n_{a}n_{b})$ per pair. In practice, the computational burden of TWED in this study is modest because the compared sequences are short respiratory interval sequences rather than long raw waveforms. Depending on the local breathing rate and the window length, each RIS typically contains only approximately 5–20 inter-breath intervals. Therefore, although the pairwise complexity of TWED is $O(n_{a}n_{b})$, the absolute cost per comparison remains limited in practice. In the present feature design, TWED is computed only between the current epoch and a fixed set of neighboring epochs and is then summarized into a small number of aggregate statistics. This makes the computation feasible for offline whole-night analysis in home-monitoring scenarios. Among the three time scales, the 390-s RIS comparisons are the most computationally demanding, but they still operate on short interval sequences and are therefore substantially less demanding than direct long-sequence waveform alignment.

The features of respiratory variability introduced in the previous section reflect the volatility indicators between respiratory intervals within an epoch. Due to individual differences, it is difficult to find a fixed respiratory sequence template that corresponds to a specific sleep stage. We know that the respiratory features of REM and NREM stages are somewhat different, and the respiration in REM stage is more irregular. Therefore, intuitively, during a single sleep session of the same subject, as sleep deepens and respiratory stability increases, the similarity of the RIS will increase accordingly. By design, smaller TWED (lower mean/median/min and often lower std) indicate more stable respiratory patterns; thus NREM epochs are expected to yield smaller aggregates than REM. A summary of all RIS features is shown in Table 3.

### 2.4 Feature normalization

After feature extraction, each feature was normalized within subject by subtracting the mean and dividing by the standard deviation of that feature calculated from the same subject's full-night feature sequence, resulting in zero mean and unit standard deviation. This subject-specific normalization used only the unlabeled feature values derived from the piezoelectric recording; no PSG-derived sleep-stage labels, manual annotations, or other ground-truth information were used when computing the normalization parameters. This design was chosen because the proposed method is intended for *post-hoc* offline analysis of a completed overnight recording, in which the full-night unlabeled piezoelectric signal of the target subject is available before normalization and classification.

### 2.5 Classifier

We used XGBoost, a gradient-boosted decision tree ensemble, to classify REM and NREM. XGBoost iteratively improves performance by fitting residuals and adding trees scaled by a learning rate. It incorporates regularization and second-order derivatives to control complexity and accelerate computation. In sleep stage classification tasks, features such as movement intensity and respiration intervals are often not linearly separable, while XGBoost can automatically handle non-linear, complex feature interactions, preventing overfitting and delivering strong performance in classification tasks.

### 2.6 Post processing

Drawing from established patterns of sleep architecture, we observed that REM stages typically demonstrate a certain degree of continuity. Therefore, we applied a rule-based post-processing method to reduce fragmentation in epoch-by-epoch REM predictions. Specifically, we defined a fusion threshold T_REM: if the number of epochs between two successive predicted REM segments was smaller than T_REM, all epochs in that interval were reassigned to REM, as illustrated in Figure 1D.

### 2.7 Experiments and evaluation

#### 2.7.1 Cross-validation

We used subject-wise leave-one-out cross-validation (LOOCV), in which one subject was held out for testing and the remaining subjects were used for model training in each outer iteration. *T_REM* was treated as a hyperparameter and selected independently within each outer LOOCV fold using only the corresponding training subjects. Specifically, for each outer fold, the training subjects were further partitioned by subject-grouped 5-fold cross-validation, and candidate values of *T_REM* were evaluated on the inner validation subjects. The value yielding the best mean validation performance was selected and then applied only to the held-out subject in the outer fold. Final performance was obtained by averaging results across all outer folds.

We also performed a subject-grouped 10-fold cross-validation analysis. Subjects were partitioned into 10 outer folds, and model training within each outer training set followed the same nested procedure as in the LOOCV evaluation, with subject-grouped 5-fold cross-validation used in the inner loop for selection of the *T_REM*.

#### 27.2 Feature evaluation

We first examined whether the body-movement features and RIS-derived features extracted in this study differed significantly between REM and NREM stages, so as to preliminarily assess their feasibility for sleep-stage classification. For each feature, a two-sided Mann–Whitney U test was applied to compare the REM and NREM distributions.

To assess feature importance within the classifier, we used XGBoost's Average Gain (AG), which quantifies the average reduction in the objective function contributed by a feature when it is used for node splitting across all trees. A larger AG indicates a greater contribution to predictive performance. In principle, feature ranking may vary across LOOCV folds because the training set differs slightly in each iteration. However, in practice, because the training cohort was relatively large (85 subjects), the feature-importance rankings were highly consistent across folds. Therefore, to evaluate the contribution of different feature subsets, we randomly selected an AG-based ranking result, incrementally added features according to that ranking, and plotted the corresponding LOOCV average Cohen's Kappa as a function of the number of included features.

In addition, to examine whether clinical heterogeneity influenced the proposed TWED-based respiratory stability features, we performed two subject-level subgroup analyses. First, subjects were grouped according to respiratory event burden (AHI < 5 vs. AHI ≥ 5). Second, within the AHI < 5 subset, subjects were further grouped as sleep-disorder-related or psychiatric/psychological-disorder-related according to the pre-monitoring diagnosis. Representative TWED features from the 30 s, 270 s, and 390 s scales were selected based on feature-importance ranking.

For each subject, representative TWED feature unnormalized values were summarized separately within REM and NREM using the median across all available epochs of the corresponding stage, and the REM–NREM difference (Δ) was calculated. To avoid pseudo-replication, all subgroup comparisons were performed using subject-level summaries rather than individual epochs. Because subgroup distributions were not assumed to be Gaussian, group comparisons were conducted using the Mann–Whitney U test. Holm correction was applied to account for multiple comparisons. Effect size was quantified using rank-biserial correlation.

#### 2.7.3 Performance metrics

We used accuracy, Cohen's Kappa, REM-specific precision, recall, and F1-score to evaluate classifier performance. In addition, weighted precision, weighted recall, and weighted F1-score were reported to summarize overall two-class performance under class imbalance. In addition to epoch-level classification metrics, we performed Bland–Altman analysis at the subject level to assess agreement between the predicted and manually scored ratio of REM to NREM (RORN).

## 3 Results

**Figure 3:**
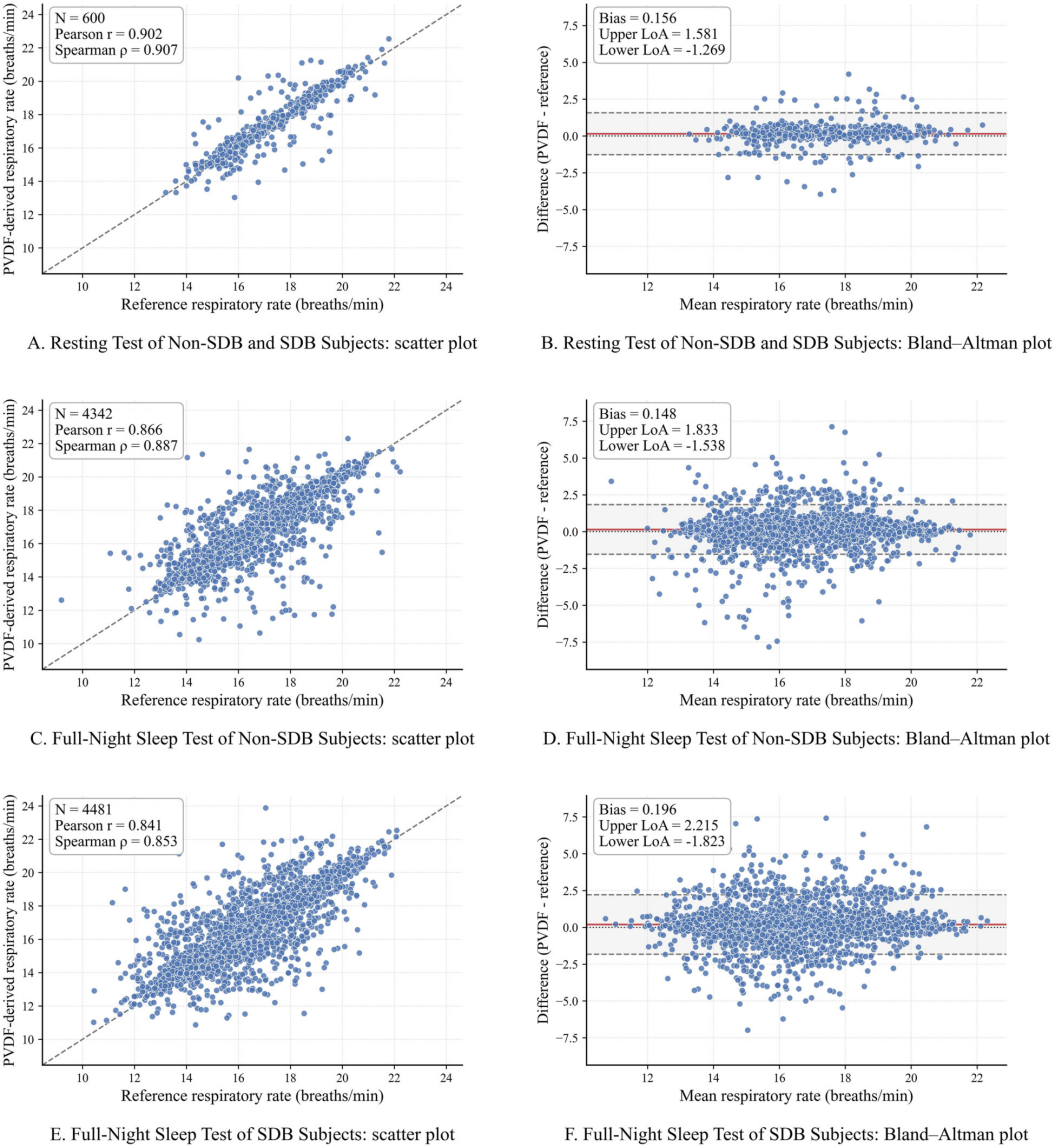
Agreement between PVDF-derived respiratory rate and the airflow reference in the respiratory tests. **(A,B)** Resting test of the 10 selected subjects. **(C,D)** Full-night sleep test in Non-SDB subjects. **(E,F)** Full-night sleep test in SDB subjects. Left panels show scatter plots, and right panels show Bland–Altman plots.

Table 4 and Table 5 summarize the results of PVDF-derived respiratory signal extraction in the resting and full-night sleep tests, and Figure 3 shows the corresponding agreement analyses against the airflow reference. In the resting test, the mean FPR, mean FNR, and mean BR30 across the 10 selected subjects were 4.31%, 2.23%, and 0.45 bpm, respectively. The corresponding agreement metrics were Pearson's r=0.902, Spearman's ρ=0.907, a bias of 0.156 breaths per minute, and limits of agreement from −1.269 to 1.581 bpm. In the full-night sleep test, the mean FPR, mean FNR, and mean BR30 were 5.33%, 2.90%, and 0.49 bpm in the Non-SDB group, and 5.78%, 3.61%, and 0.61 bpm in the SDB group, respectively. For the Non-SDB group, agreement analysis yielded Pearson's r=0.866, Spearman's ρ=0.887, a bias of 0.148 bpm, and limits of agreement from −1.538to 1.833 bpm. For the SDB group, Pearson's , *r* = 0.841Spearman's ρ=0.853, a bias of 0.196 breaths per minute, and limits of agreement from −1.823 to 2.215 bpm. These findings show that PVDF-derived respiratory signals were consistently extracted with low detection error and good agreement with the airflow reference under both controlled posture changes and overnight sleep conditions.

**Table 4:** Performance results of resting test.

Subject	1	2	3	4	5	6	7	8	9	10	Mean
Dur (min)	30	30	30	30	30	30	30	30	30	30	**30**
FPR (%)	3.54	4.94	5.49	4.41	1.94	2.69	4.12	4.2	6.7	5.09	**4**.**31**
FNR (%)	1.2	1.83	1.93	2.94	1.04	2.46	4.37	2.32	1.76	2.45	**2**.**23**
BR30 (bpm)	0.29	0.43	0.3	0.53	0.37	0.64	0.59	0.51	0.51	0.38	**0**.**45**

The bolded values in the table represent the mean values for that indicator across the 10 participants.

**Table 5:** Performance results of full-night sleep test.

Subject	Non-SDB Subjects						SDB Subjects					
	1	2	3	4	5	Mean	6	7	8	9	10	Mean
Dur (min)	406	499.5	349	457	459.5		412.5	447	464.5	435	481.5	
FPR (%)	4.35	6.42	6.66	5.36	3.86	**5**.**33**	5.25	6.98	3.82	6.68	6.17	**5**.**78**
FNR (%)	2.24	3.22	2.82	3.08	3.12	**2**.**9**	3.41	5.57	2.78	2.77	3.51	**3**.**61**
BR30 (bpm)	0.39	0.48	0.46	0.55	0.58	**0**.**49**	0.63	0.69	0.54	0.62	0.56	**0**.**61**

Bpm, breaths per minute.

The bolded values in the table represent the mean values for that indicator across the 10 participants.

Table 6 reports the performance results of nested LOOCV under different feature sets and with or without normalization conditions. On the one hand, we observe that applying feature normalization significantly improves REM/NREM classification performance for all feature sets. It also indicates that, to some extent, this procedure helps mitigate between-subject variability in respiratory physiology, as reflected by the reduced standard deviations of the performance metrics. On the other hand, after normalization，when using RV combined with either BM or RIS_S as the baseline feature set, adding the remaining feature set (BM or RIS_S, respectively) consistently leads to a significant performance gain. In particular, when the TWED-based RIS similarity features were added to the normalized conventional BM+RV feature set, accuracy increased from 82.55 ± 14.05 to 84.39 ± 12.76, Cohen's Kappa increased from 0.478 ± 0.261 to 0.524 ± 0.241, and the REM F1-score increased from 0.562 ± 0.235 to 0.603 ± 0.211, indicating that the proposed similarity features, when used in conjunction with conventional feature sets, not only improves overall classification performance but also enhances REM discriminative power in cases of class imbalance. The six pooled confusion matrices across outer LOOCV folds are provided in the Supplementary Appendix Figure S7. Supplementary Appendix Table S1 reports a similar trend in nested 10-fold cross-validation, and its results also demonstrate the improvement in REM/NREM discrimination made by the proposed similarity features when combined with conventional features.

**Table 6:** Summary of REM/NREM classification performance (nested LOOCV) for different feature sets, with and without feature normalization.

Feature Set	BM+RV	RV+RIS_S	BM+RV+RIS_S	BM+RV	RV+RIS_S	BM+RV+RIS_S
Normalization	No	No	No	Yes	Yes	Yes
Acc (%)	80.56 ± 16.38^NS^	81.07 ± 15.98^NS^	81.29 ± 15.14	82.55 ± 14.05*	83.31 ± 12.66^NS^	**84.39** ± **12.76**
Kappa	0.421 ± 0.278*	0.46 ± 0.271^NS^	0.447 ± 0.281	0.478 ± 0.261*	0.497 ± 0.247*	**0.524** ± **0.241**
REM Precision	0.522 ± 0.296*	0.547 ± 0.297^NS^	0.552 ± 0.299	0.57 ± 0.275*	0.561 ± 0.268*	**0.6** ± **0.21**
REM Recall	0.68 ± 0.291^NS^	0.716 ± 0.284^NS^	0.687 ± 0.307	0.703 ± 0.262*	0.736 ± 0.238^NS^	**0.735** ± **0.226**
REM F1-score	0.512 ± 0.263*	0.545 ± 0.25^NS^	0.533 ± 0.258	0.562 ± 0.235*	0.579 ± 0.223*	**0.603** ± **0.211**
Weighted Precision	0.887 ± 0.076^NS^	0.891 ± 0.068^NS^	0.888 ± 0.074	0.889 ± 0.07^NS^	0.891 ± 0.072^NS^	**0.897** ± **0.069**
Weighted Recall	0.806 ± 0.164^NS^	0.811 ± 0.16^NS^	0.813 ± 0.151	0.826 ± 0.14*	0.833 ± 0.127^NS^	**0.844** ± **0.128**
Weighted F1-score	0.816 ± 0.152^NS^	0.82 ± 0.147^NS^	0.822 ± 0.139	0.835 ± 0.126*	0.844 ± 0.114^NS^	**0.853** ± **0.113**

Acc, Accuracy; Kappa, Cohen's Kappa; BM, Body-movement; RV, respiratory variability; RIS_S, RIS similarity.

Significance of difference between the results obtained using feature set of this column and “BM+RV+RIS_S” was examined with a paired two-sided Wilcoxon signed-rank test (*p < 0.05, NS: not significant). For all metrics, significant difference was found between the results obtained with and without feature normalization at *p* < 0.05.

The bolded values in the table represent the overall best results of the REM/NREM classification task (nested LOOCV).

It should be noted that in all experiments in Table 6 and Supplementary Appendix Table S1, T_REM was selected independently within each outer fold using only the corresponding training subjects through inner subject-wise GroupKFold (5-fold) validation over candidate values ranging from 0 to 40 epochs in steps of 2. Supplementary Appendix Figure S3 summarizes the distribution of the selected T_REM values across outer folds in the experiment of “*BM+RV+RIS_S* with normalization”.

**Figure 4:**
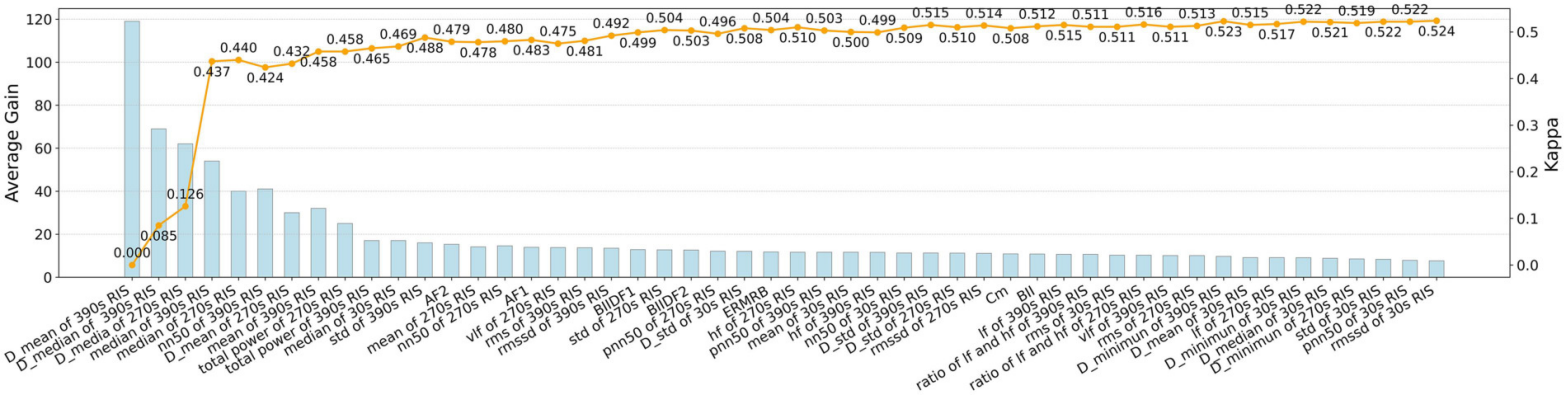
Feature importance ranking based on AG and the LOOCV average Cohen's kappa obtained when selecting the top N (*N* ≤ 50) features.

The feature importance ranking of all 50 features based on AG is presented in Figure 4. The bar graph in this figure represents the ranking of each feature's discriminative ability in distinguishing between REM and NREM stages. It is evident that among the top-ranked RIS features, the majority are derived from the 270-second and 390-second RIS (In particular, the three most important features are RIS similarity features). This further underscores that, in sleep stage classification, longer temporal contexts tend to outperform shorter ones. To evaluate the contribution of each feature, we utilized the AG-based feature importance ranking, starting with the single most important feature and then incrementally adding one feature at a time. For each of the resulting 50 feature subsets, we computed the nested LOOCV average Cohen's Kappa and plotted the corresponding line-plot in Figure 4. The best classification result was achieved using the combination of the all 50 features, which yielded an average Cohen's Kappa of 0.524. Supplementary Appendix Figure S2 shows the vast majority of RIS features exhibit significant differences between REM and NREM stages. Supplementary Appendix Figures S4A,B present, for two subjects over an entire night of monitoring, the comparison between manual sleep-stage labels and algorithm-predicted labels (with WAKE stages omitted). Each figure also illustrates the temporal trends of the two RIS similarity features—$D_{median}$ of the 390s RIS in one case and $D_{mean}$ of the 390s RIS in the other. As can be seen, when the subject enters REM sleep, the TWED between RIS increases markedly, which thereby aids in distinguishing REM and NREM stages.

For the AHI-based subgroup analyses (Table 7; Supplementary Appendix Figure S5), three representative TWED features were selected from the 30s, 270s, and 390s scales based on feature-importance ranking. Although the median values showed a directionally consistent pattern, with the AHI ≥ 5 group tending to exhibit higher NREM TWED values and smaller REM–NREM differences than the AHI < 5 group, none of the comparisons remained statistically significant after Holm correction. The strongest trend was observed for the TWED of the 30-s variability feature (Holm-corrected *p* = 0.095, rank-biserial = −0.360). Overall, these results do not provide robust evidence that AHI grouping was associated with a clear shift in the representative TWED-based respiratory stability features in the current sample.

**Table 7:** Exploratory subject-level comparison of representative TWED-based respiratory stability features between the AHI < 5 and AHI ≥ 5 groups.

Feature	AHI < 5 (*n* = 63)	AHI ≥ 5 (*n* = 20)	Holm-corrected *p*	Rank-biserial
$D_{mean}$ of 390s RIS (NREM)	28,899.19 [24,750.81, 39,194.03]	33,133.52 [27,760.38, 37,588.42]	0.813	0.163
$D_{mean}$ of 390s RIS (Δ)	14,276.47 [6,811.73, 21,377.65]	11,375.31 [4,721.30, 16,372.42]	0.813	−0.190
$D_{median}$ of 270s RIS (NREM)	22,518.95 [19,464.13, 30,627.07]	25,769.05 [21,791.79, 30,396.01]	0.813	0.167
$D_{median}$ of 270s RIS (Δ)	11,122.92 [5,017.28, 16,270.89]	8,696.93 [3,771.33, 11,279.77]	0.731	−0.217
$D_{std}$ of 30s RIS (NREM)	1,223.16 [1,133.14, 1,522.85]	1,339.53 [1,172.13, 1,492.23]	0.813	0.122
$D_{std}$ of 30s RIS (Δ)	315.56 [108.76, 520.85]	208.60 [83.56, 287.03]	0.095	−0.360

Data are presented as median [Q1, Q3]. Δ denotes the subject-level REM–NREM difference. Only subjects with at least 5 REM epochs and 20 NREM epochs were included in this analysis. The corresponding boxplots are provided in Appendix Figure S5.

For the diagnosis-based subgroup analyses within the AHI < 5 subset (Table 8; Supplementary Appendix Figure S6), most representative TWED features did not differ significantly between sleep-disorder-related and psychiatric/psychological-disorder-related subjects after multiple-testing correction. One longer-scale REM–NREM difference feature, the $D_{median}$ of 270s RIS (Δ), showed a Holm-corrected *p*-value of 0.048 (rank-biserial = −0.416), whereas the remaining comparisons were not statistically significant. Given that this result was isolated within a small exploratory subgroup analysis and was not accompanied by a broader consistent pattern across features, it should be interpreted cautiously. Taken together, these findings do not support a robust association between the examined diagnosis grouping and the TWED-based respiratory stability features in the current dataset.

**Table 8:** Exploratory subject-level comparison of representative TWED-based respiratory stability features between sleep-disorder-related and psychiatric/psychological-disorder-related subjects within the AHI < 5 subset.

Feature	Sleep-related (*n* = 23)	Psychiatric/psychological (*n* = 35)	Holm-corrected *p*	Rank-biserial
$D_{mean}$ of 390s RIS (NREM)	30,721.19 [25,603.95, 36,594.87]	28,899.19 [24,818.10, 43,146.77]	1.000	−0.021
$D_{mean}$ of 390s RIS (Δ)	18,716.58 [11,488.05, 24,454.42]	12,877.50 [4,375.13, 17,738.90]	0.075	−0.381
$D_{median}$ of 270s RIS (NREM)	23,830.84 [20,047.20, 28,910.51]	22,518.95 [19,580.35, 33,335.15]	1.000	−0.041
$D_{median}$ of 270s RIS (Δ)	14,945.27 [8,887.60, 18,499.54]	8,633.38 [3,357.63, 13,429.05]	0.048	−0.416
$D_{std}$ of 30s RIS (NREM)	1,237.98 [1,154.27, 1,553.87]	1,215.31 [1,118.37, 1,508.17]	1.000	−0.061
$D_{std}$ of 30s RIS (Δ)	312.63 [82.71, 525.00]	310.47 [119.29, 472.58]	1.000	−0.024

Data are presented as median [Q1, Q3]. Δ denotes the subject-level REM–NREM difference. Only subjects with at least 5 REM epochs and 20 NREM epochs were included in this analysis. The corresponding boxplots are provided in Supplementary Appendix Figure S6.

**Figure 5:**
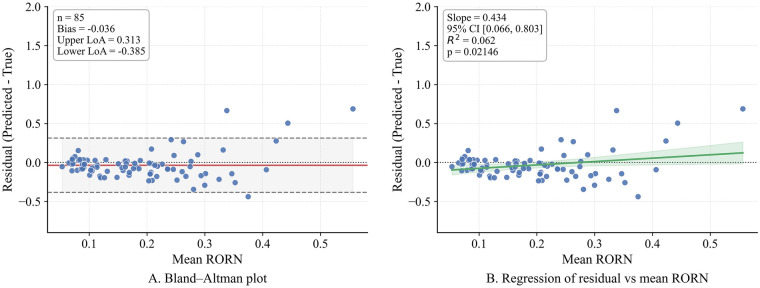
Bland–altman analysis of predicted versus manually scored RORN across 85 subjects. **(A)** Bland–Altman plot showing the mean bias and 95% limits of agreement. **(B)** Linear regression of the Bland–Altman residuals (predicted RORN minus manually scored RORN) against the corresponding mean RORN, with the fitted regression line and 95% confidence interval.**s.**

To further evaluate subject-level agreement, we performed Bland–Altman analysis on the RORN across all 85 subjects (Figure 5). The predicted RORN values were obtained from the subject-wise nested LOOCV. The mean bias was −0.036, indicating only minimal overall underestimation of RORN by the proposed method. The 95% limits of agreement ranged from −0.385 to 0.313. To formally assess proportional bias, we regressed the Bland–Altman residuals (predicted RORN minus manually scored RORN) against the corresponding mean RORN values. The fitted slope was 0.434 (95% CI 0.066–0.803), with an R² of 0.062 and a *p*-value of 0.021. These results indicate a statistically significant but weak positive proportional bias, suggesting that estimation error tended to increase modestly as mean RORN increased.

Table 9 compares the performance of our proposed REM/NREM classification method with those reported in the literature. For example, Domingues et al. (22) achieved 79% accuracy and a Cohen's Kappa of 0.58 in their study; although their Cohen's Kappa exceeds ours, their approach relied on three signal modalities—ECG, body-movement, and respiration—collected by three different sensors, which limits its convenience. In the results of Jaworski et al. (16), the Cohen's Kappa was slightly higher than in our study, but they also incorporated vital-sign parameters provided by the Apple Watch and Ballistocardiography sensor, and their number of recruited subjects was much smaller than ours. When considering single-device monitoring, our method showed competitive performance relative to other studies—for example, Singh et al. (23) achieved a Cohen's Kappa of 0.48 using ECG signals, and Walch et al. (15) reported 72% accuracy with the Apple Watch.

**Table 9:** Comparison of REM/NREM classification results with those reported in literature.

Author, Year	Signal modalities	Number of subjects	Number of features	Accuracy	Cohen's Kappa
Domingues et al. 2014 (22)	ECG, Act, RIP	20	24	79%	0.58
Singh et al. 2016 (23)	ECG	20	9	72.8%	0.48
Walch et al. 2019 (15)	Apple Watch^a^	39	3	72%	-
Jaworski et al. 2024 (16)	BCG, Apple Watch^b^	6	130	72.7%	0.534
Our work (nested LOOCV)	Piezoelectric signals	85	50	84.39%	0.524
Our work (nested 10-fold cross-validation)	Piezoelectric signals	85	50	83.56%	0.502

RIP, respiratory inductance plethysmography; Act, Actigraphy; BCG, Ballistocardiography.

a Walch et al. (15) utilized vital-sign parameters provided by the Apple Watch to estimate activity counts, heart-rate standard deviation, and time-based features.

b Jaworski et al. (16) used vital-sign parameters output by the Apple Watch to estimate heart rate variability.

## 4 Discussion

Using 30-s epochs from 85 clinical participants and subject-wise LOOCV with XGBoost, our model achieved an average accuracy of 84.39% and a Cohen's Kappa of 0.524 for REM/NREM discrimination. Relative to using RIS features alone, adding body-movement features improved performance, underscoring their complementary value. We further introduced TWED-based RIS similarity: for each target epoch, we computed TWED between its respiratory interval sequence and those of neighboring epochs and summarized the distances by mean, median, standard deviation, and minimum. Physiologically, deeper sleep is characterized by more regular autonomic control of breathing, yielding higher short-term similarity, whereas REM exhibits transient irregularity due to dreaming, altered muscle tone, and fluctuations in respiratory control. Supplementary Appendix Figure S4 demonstrates that these similarity features capture the contrast between irregular REM respiration and more stable NREM respiration.

Feature-importance analysis (Figure 4) indicates that longer sequences (270 s and 390 s) dominate the top ranks, suggesting that extended temporal context enhances respiration-based staging by averaging slow trends and attenuating low-frequency drift and sporadic disturbances. To assess the potential influence of cohort heterogeneity on the proposed TWED-based respiratory stability features, we conducted subject-level subgroup analyses based on AHI and diagnosis grouping. Although the AHI ≥ 5 group showed a directional tendency toward higher NREM TWED values and smaller REM–NREM differences, none of these effects remained significant after multiple-comparison correction. Likewise, within the AHI < 5 subset, most diagnosis-based comparisons were not significant, and the single corrected *p*-value below 0.05 was isolated rather than part of a consistent pattern. These findings therefore remain descriptive and exploratory, rather than supporting a robust association between clinical subgroup definitions and TWED-based respiratory stability features. Because subgroup sizes were limited and no healthy controls were included, stronger conclusions about diagnosis-related effects relative to a healthy baseline cannot be drawn. Future studies with larger, more balanced, and healthier cohorts are needed.

Previous studies have used combinations of ECG (24), wearable photoplethysmography (25), accelerometry (26), or ballistocardiography (16) to perform REM/NREM or broader sleep-stage classification. Many of these multimodal approaches benefit from richer physiological information but also increase instrumentation burden and reduce convenience for repeated or long-term use. In contrast, our approach uses a single under-mattress piezoelectric sensor and derives both respiratory and movement-related information from the same unobtrusive signal source. This is an important practical advantage for home-like deployment. Nevertheless, comparisons across studies should be interpreted cautiously. The studies summarized in Table 9 differ substantially in cohort size, subject composition, sensing modality, feature design, task definition, and evaluation protocol. Therefore, Table 9 is intended to position the proposed method within the literature rather than to imply a direct ranking across heterogeneous experimental settings.

The present study was intentionally formulated as a binary REM/NREM classification problem because its primary objective was not full-stage sleep scoring, but to test whether respiratory pattern stability could improve discrimination between the two major sleep states within sleep itself. In this sense, the proposed TWED-based RIS similarity features were designed to capture within-sleep differences in respiratory temporal organization rather than to characterize the full spectrum of nocturnal states. Accordingly, the findings should be interpreted primarily as evidence that respiratory pattern stability provides useful complementary information for REM/NREM discrimination, rather than as evidence that the present framework is already suitable for complete sleep staging.

From an application perspective, the proposed method can be considered a low-burden adjunctive approach for *post-hoc* whole-night analysis and unobtrusive longitudinal monitoring of sleep architecture in home-like settings. Potential use-cases include analyzing completed overnight recordings to track night-to-night changes in REM/NREM balance, monitoring sleep-pattern trends over time, and supporting repeated follow-up during behavioural or pharmacological interventions when full PSG is impractical. It may also be useful in sleep health management scenarios requiring frequent measurements with minimal instrumentation. However, at its current level of performance, with its present binary REM/NREM scope and full-night subject-specific normalization design, the method should be regarded as a complementary offline home-monitoring approach rather than a replacement for PSG-based clinical diagnosis, real-time sleep staging, or full sleep-stage scoring.

This study has several limitations. First, all participants were recruited from a clinical cohort, including subjects with respiratory and psychiatric disorders, and no healthy control group was included. This may limit generalizability to broader home-monitoring populations. Future work should therefore include larger, more balanced cohorts with healthy controls to clarify whether disorder-specific effects on respiratory stability features are present and reproducible. Second, the Bland–Altman analysis showed minimal average bias in subject-level RORN estimation, but also a weak yet statistically significant proportional bias, suggesting that estimation error was not fully uniform across the RORN range and may be larger in subjects with higher RORN. Further model refinement or calibration may therefore be beneficial. Third, the present study was restricted to binary REM/NREM classification and did not model WAKE. Although this design was motivated by within-sleep differences in respiratory pattern stability, quiet wakefulness may be misclassified as sleep in practical use, especially during WASO. Future work will develop a dedicated sleep/wake classifier and integrate it with the present REM/NREM model in a hierarchical framework.

## 5 Conclusions

In this study, we examined whether respiratory pattern stability derived from piezoelectric signals could improve discrimination between REM and NREM sleep. To this end, we integrated conventional body-movement and respiratory variability features with TWED-based RIS similarity features and evaluated an XGBoost classifier under a nested cross-validation framework. The results suggest that TWED-based RIS similarity features provide useful complementary information beyond conventional body-movement and respiratory descriptors and improve REM/NREM discrimination. Overall, these findings support the feasibility of using respiratory pattern stability derived from non-contact piezoelectric signals for within-sleep REM/NREM classification. At the current level of performance, the proposed method is better interpreted as an offline whole-night analysis approach for completed overnight recordings and longitudinal trend assessment in home-like settings, rather than as a stand-alone tool for clinical diagnosis. Further validation in broader populations and more comprehensive staging tasks is still needed.

## Data Availability

The raw data supporting the conclusions of this article will be made available by the authors, without undue reservation.
