# Supplementary material for: Non-contact REM/NREM sleep staging from piezoelectric signals using respiratory and body-movement features with auxiliary TWED-based respiratory stability measures
Source: Front Digit Health. 2026 Jun 15;8:1780166. doi: 10.3389/fdgth.2026.1780166 (PMC13310895; doi:10.3389/fdgth.2026.1780166)
Supplement: Supplementary file 1 [file Supplementaryfile1.docx]

# **Supplementary Material**


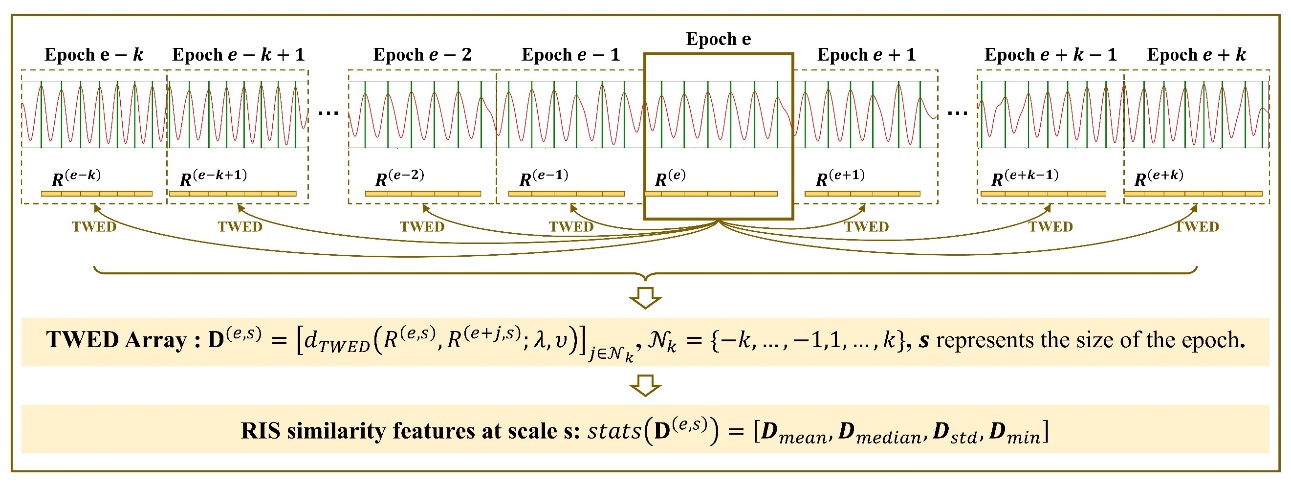


**Figure S1.** The process of extracting RIS similarity features for a 30-s epoch example.


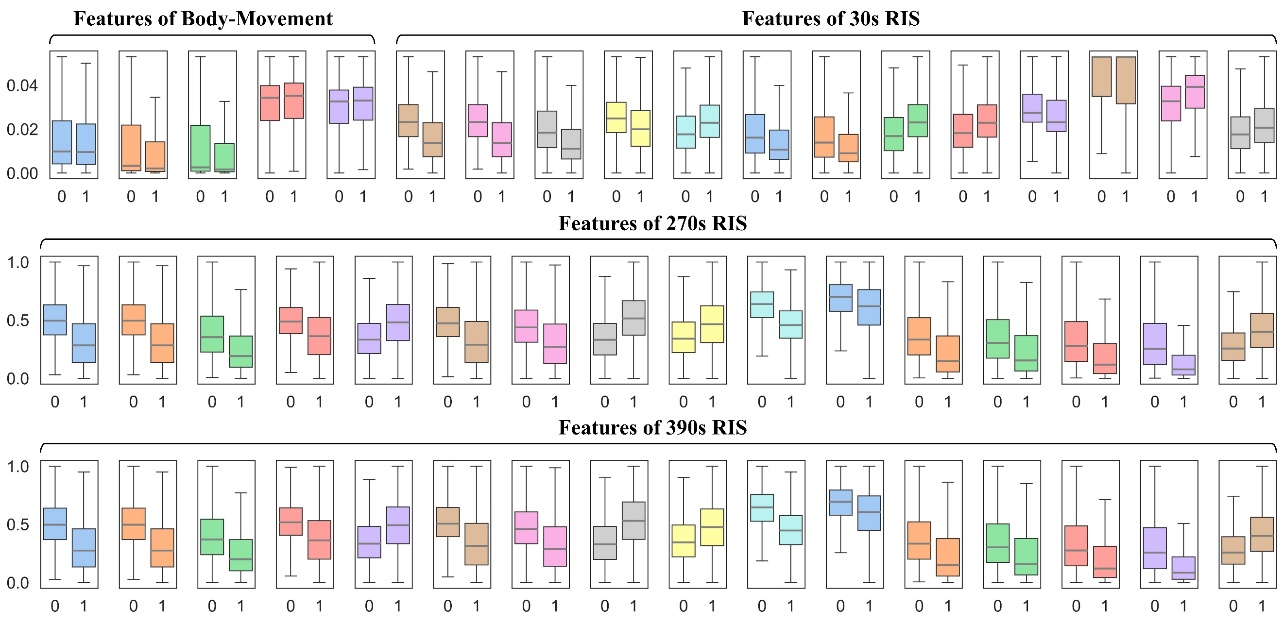


**Figure S2.** Boxplots of body-movement and RIS features, where 0 represents REM and 1 represents NREM. Features were normalized and outliers removed before plotting. Unpaired Mann–Whitney tests indicate that all features differ significantly between the two classes (p < 0.01).


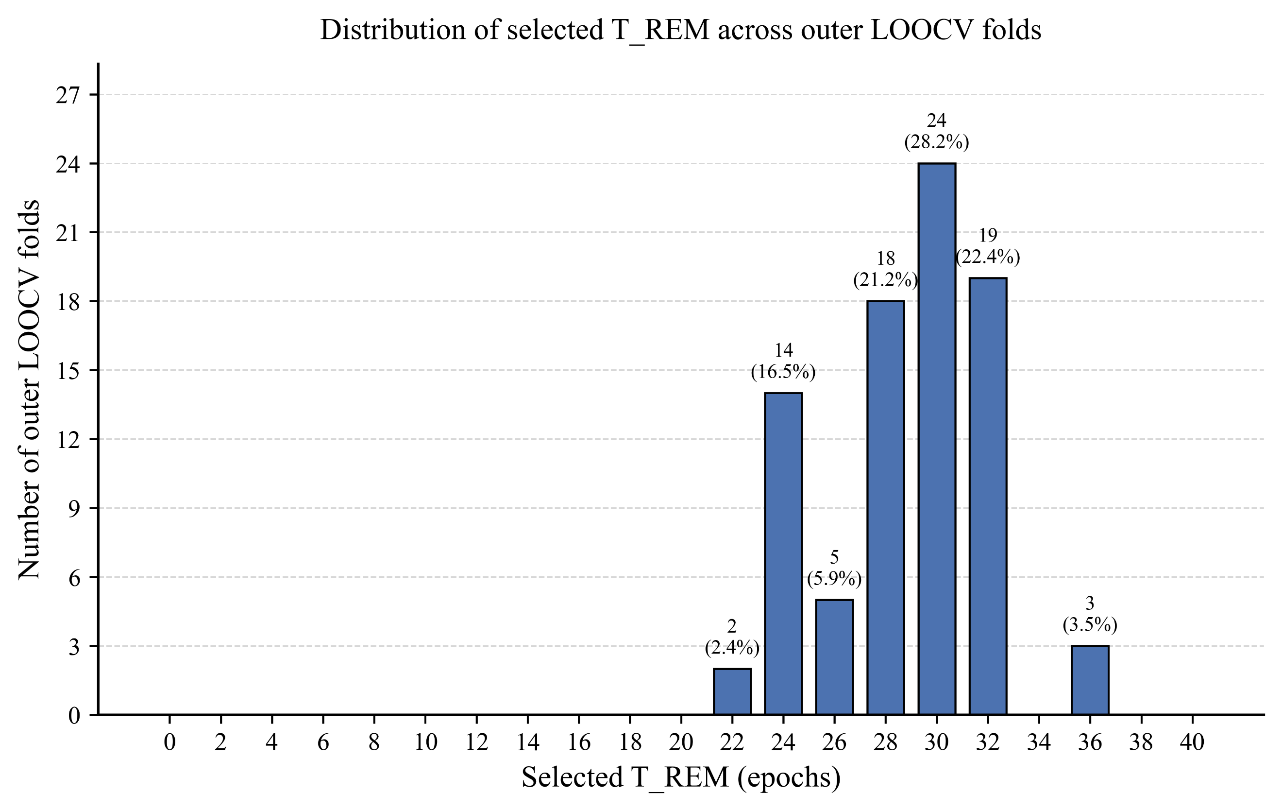
**Figure S3.** Distribution of the fold-specific $T\_REM$ selected across the outer leave-one-out cross-validation (LOOCV) folds. In each outer fold, $T\_REM$ was determined using only the corresponding training subjects through inner subject-wise GroupKFold (5-fold) validation over candidate values ranging from 0 to 40 epochs in steps of 2.


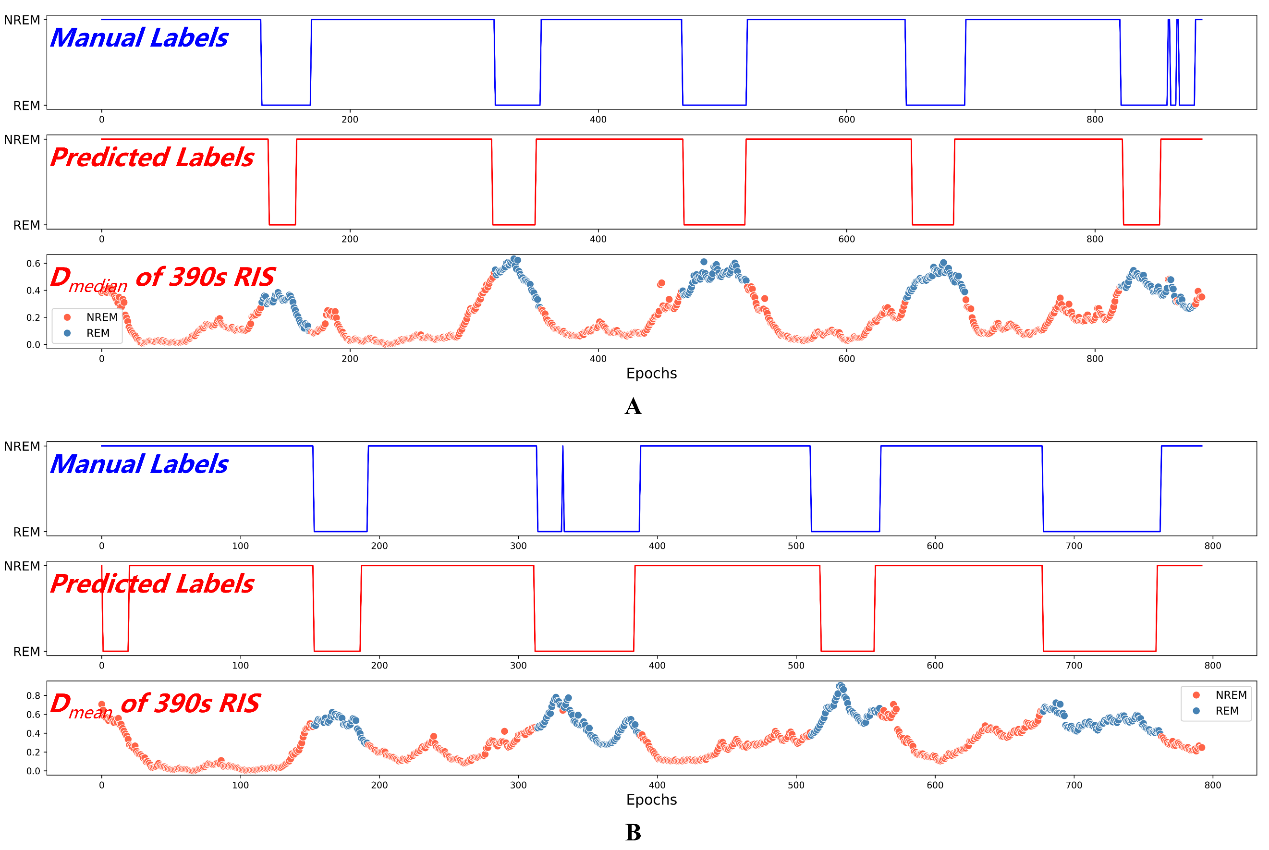


**Figure S4.** **A**. Temporal trend of “$\boldsymbol{D}_{median}$ of 390s RIS” compared with manual and predicted sleep‐stage labels (from an all-night recording of a 49-year-old female subject). **B.** Temporal trend of “$\boldsymbol{D}_{mean}$ of 390s RIS” compared with manual and predicted sleep-stage labels (from an all-night recording of a 21-year-old female subject). WAKE epochs were removed, so time is discontinuous; the x-axis therefore represents epoch indices.


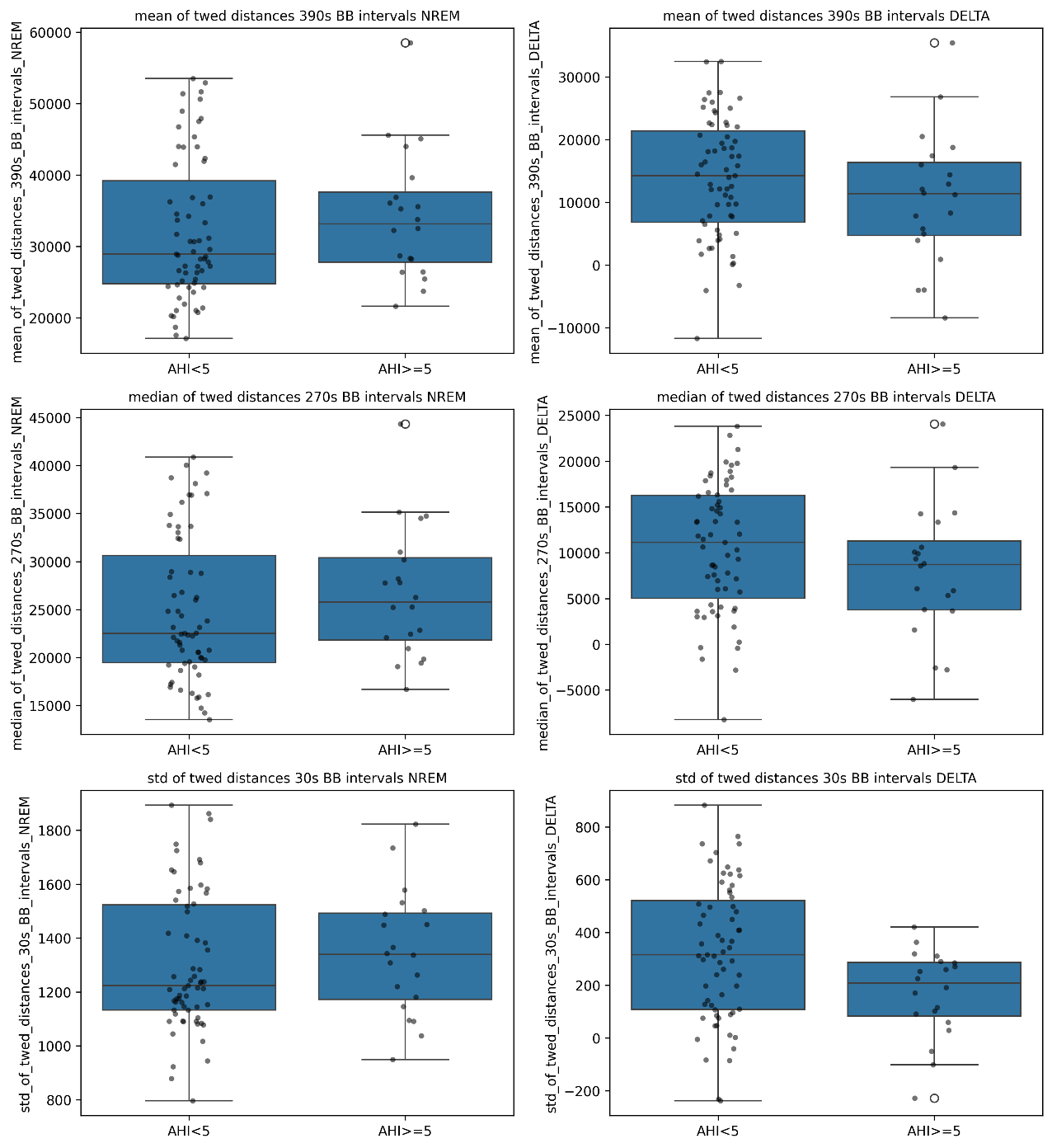
**Figure S5.** Boxplots of representative TWED-based respiratory stability features in the AHI < 5 and AHI ≥ 5 groups.


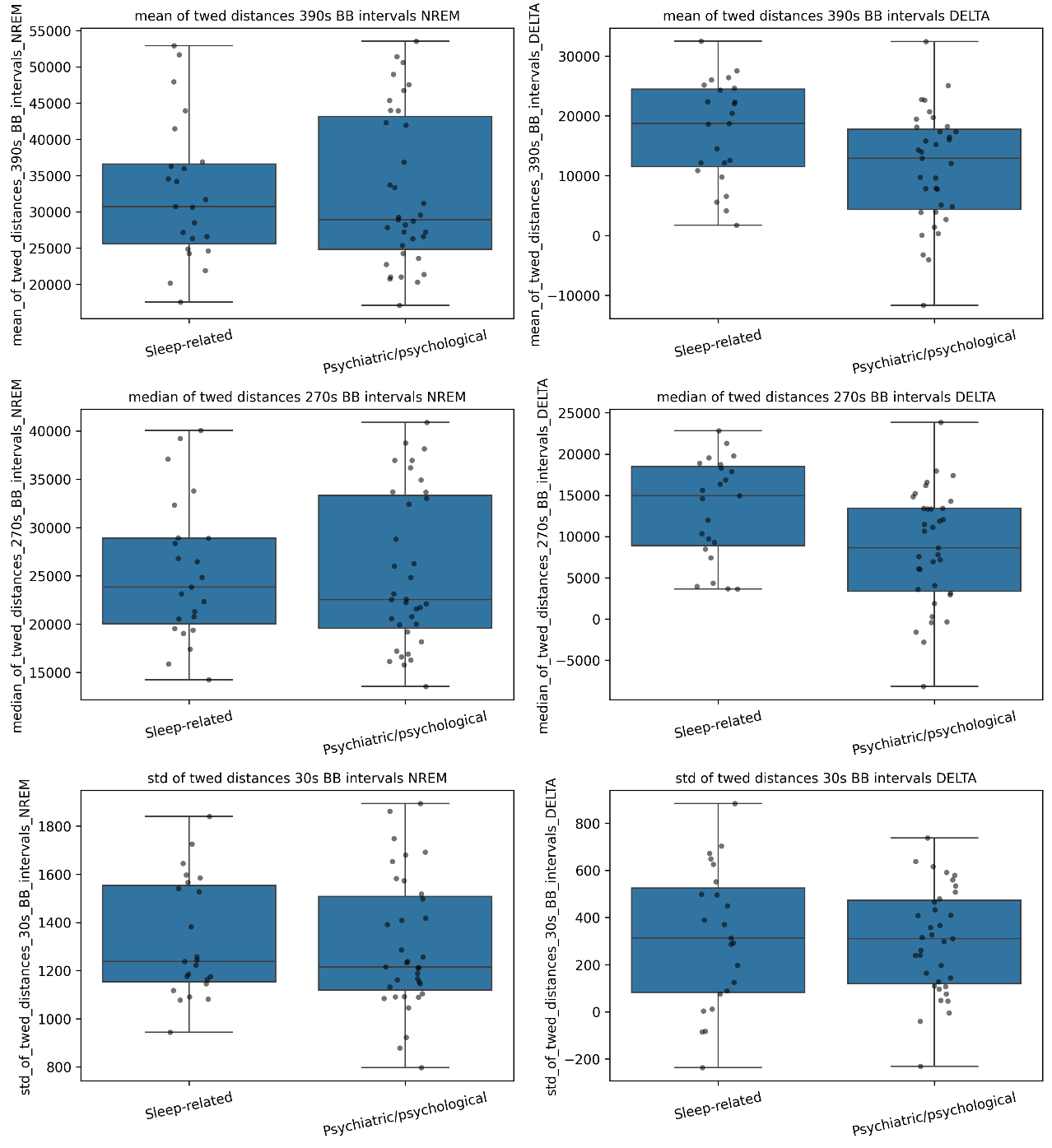
**Figure S6.** Boxplots of representative TWED-based respiratory stability features in the sleep-disorder-related and psychiatric/psychological-disorder-related groups within the AHI < 5 subset.


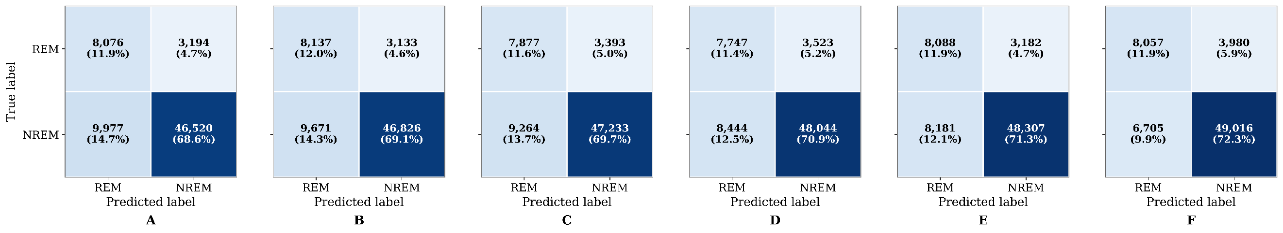


**Figure S7**. Pooled confusion matrices across all outer LOOCV folds for the six feature-set/normalization combinations shown in **Table 6**. Panels (**A–C**) correspond to $BM + RV$, $RV + RIS\_S$, and $BM + RV + RIS\_S$ without feature normalization, respectively. Panels (**D–F**) correspond to the same three feature combinations with feature normalization. Rows indicate manual labels and columns indicate predicted labels.

**Table S1.** Summary of REM/NREM classification performance (nested 10-fold cross-validation) for different feature sets, with and without feature normalization.

| **Feature Set** | $BM+RV$ | $RV+RIS\_S$ | $BM+RV+RIS\_S$ | $BM+RV$ | $RV+RIS\_S$ | $\boldsymbol{BM+RV+RIS\_S}$ |
| --- | --- | --- | --- | --- | --- | --- |
| **Normalization** | No | No | No | Yes | Yes | **Yes** |
| **Acc (%)** | 80.41 | 81.22 | 81.53 | 82.73 | 83.95 | **83.56** |
| **Kappa** | 0.432 | 0.457 | 0.451 | 0.472 | 0.491 | **0.502** |
| **REM Precision** | 0.468 | 0.479 | 0.485 | 0.508 | 0.512 | **0.524** |
| **REM Recall** | 0.664 | 0.698 | 0.695 | 0.687 | 0.715 | **0.723** |
| **REM F1-score** | 0.544 | 0.565 | 0.559 | 0.573 | 0.595 | **0.6** |
| **Weighted Precision** | 0.835 | 0.852 | 0.85 | 0.853 | 0.861 | **0.872** |
| **Weighted Recall** | 0.794 | 0.812 | 0.815 | 0.813 | 0.82 | **0.836** |
| **Weighted F1-score** | 0.804 | 0.82 | 0.82 | 0.819 | 0.832 | **0.847** |

**Acc = Accuracy, Kappa = Cohen’s Kappa,** $BM$ = Body-movement; $RV$ = respiratory variability; $RIS\_S$ = RIS similarity
